# Developmental Changes in the Association Between Cognitive Control and Anxiety

**DOI:** 10.1007/s10578-021-01150-5

**Published:** 2021-03-18

**Authors:** Courtney A. Filippi, Anni Subar, Sanjana Ravi, Sara Haas, Sonya V. Troller-Renfree, Nathan A. Fox, Ellen Leibenluft, Daniel S. Pine

**Affiliations:** 1 Emotion and Development Branch, National Institute of Mental Health, Bethesda, MD 20892 USA; 2 Department of Psychology, University of Denver, Denver, CO 80208 USA; 3 Department of Human Development and Quantitative Methodology, University of Maryland, College Park, Maryland, 20742 USA; 4 Department of Psychology, Uppsala University, Uppsala, Sweden; 5 Department of Biobehavioral Sciences, Teachers College, Columbia University, New York, NY 10027 USA

**Keywords:** AX-CPT, Cognitive control, Anxiety, Childhood, Reactive control, Proactive control

## Abstract

Anxiety has been associated with reliance on reactive (stimulus-driven/reflexive) control strategies in response to conflict. However, this conclusion rests primarily on indirect evidence. Few studies utilize tasks that dissociate the use of reactive (‘just in time’) vs. proactive (anticipatory/preparatory) cognitive control strategies in response to conflict, and none examine children diagnosed with anxiety. The current study utilizes the AX-CPT, which dissociates these two types of cognitive control, to examine cognitive control in youth (ages 8–18) with and without an anxiety diagnosis (*n* = 56). Results illustrate that planful behavior, consistent with using a proactive strategy, varies by both age and anxiety symptoms. Young children (ages 8–12 years) with high anxiety exhibit significantly less planful behavior than similarly-aged children with low anxiety. These findings highlight the importance of considering how maturation influences relations between anxiety and performance on cognitive-control tasks and have implications for understanding the pathophysiology of anxiety in children.

**Supplementary Information:**

The online version contains supplementary material available at 10.1007/s10578-021-01150-5.

## Introduction

Anxiety may impact one’s ability to control their behavior [1–4]. This could be due to differences in cognitive control abilities—anxious individuals exhibit worse working memory, inhibition, and shifting abilities when compared to non-anxious individuals [4]. However, new work suggests that anxious individuals differ from healthy individuals in their deployment of these cognitive control skills [5]. Worse performance on cognitive control tasks has been thought to reflect anxious individuals’ greater reliance on *reactive* control [1, 2], i.e., control instantiated reflexively, ‘just in time’, or in response to a stimulus (e.g., finishing a class assignment as the teacher is collecting it). This contrasts with *proactive* control, which refers to control instantiated through the maintenance of goal-relevant information (e.g., completing a class assignment well in advance). However, more research in this area is needed since relatively few studies on associations between cognitive control and anxiety utilize tasks that dissociate reactive and proactive control. Differentiating these cognitive control strategies is critical for uncovering the precise nature of cognitive control impairments in anxiety. This is particularly important in young children, who manifest immature cognitive control. To address these gaps, the current study examines performance on the AX-Continuous Performance Task (AX-CPT [6]), a task which dissociates reactive and proactive control, in children (ages 8–18) with and without anxiety disorders.

Anxiety impacts cognitive processes, which may disrupt maintenance of task goals—presumably leading to greater reliance on reactive control. However, few studies of anxiety dissociate proactive and reactive control. While several relevant studies examine control processes during conflict [4, 7–11], these studies typically rely on assessments, such as the Flanker or Go-NoGo task, which measure reactive control. In their standard forms, such tasks require participants to respond to stimuli in the absence of pre-stimulus information that can facilitate planful responding. Such tasks fail to fully engage forms of preparatory, proactive control. In contrast, other tasks, such as the AX-CPT, dissociate proactive and reactive control use. These tasks provide information that allows the participant to anticipate the need to engage control. In everyday life, we execute both proactive and reactive control strategies depending on situational factors [12] (e.g., whether you have information relevant to the upcoming decision or can retrieve relevant information fast enough to use it to prepare a response).

**Fig. 1:**
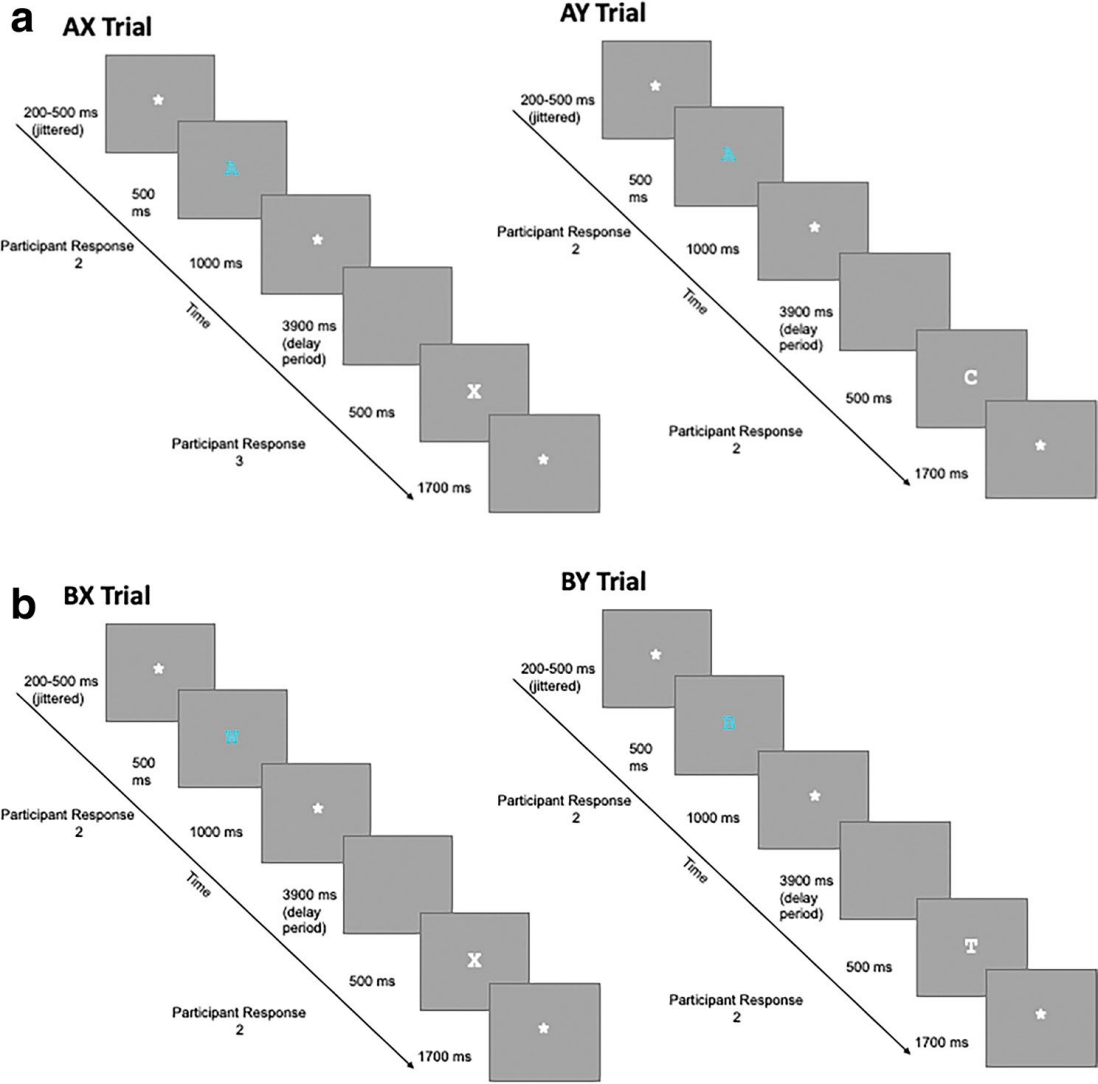
Illustrates the timing of all trials and provides a comparison of trial types. Trial types include: AX and AY (depicted in 1**a**) and BX and BY (depicted in 1**b**)

The AX-CPT evaluates the relative use of proactive and reactive control by presenting two letter pairs and asking participants to identify the pair “A–X”. The A–X pair is presented frequently, allowing the participant to develop the expectation that an A will be followed by an X. However, 10% of the time the X does not follow an A. These trials (deemed A-Y trial types; See Fig. 1) require the individual who proactively anticipates that an X will follow an A, to rapidly update their response. In contrast, other trial types provide information that consistently illustrates that the A–X pair is not present (e.g., trials where a non-A is presented first indicating that the A is missing from the A–X pair—See Fig. 1 BX and BY trials). With this design, the task presents information that helps the individual predict the response that likely will be required in the future. This task design mirrors situations where individuals use context to anticipate interference prior to executing the required behavioral response (i.e., allowing the participant to execute proactive control). By comparing accuracy and reaction time across trial types, the AX-CPT can index the degree to which an individual relies on proactive or reactive control while completing the task. At present, the only study to use the AX-CPT with anxiety-disorder patients found enhanced proactive control in treatment-seeking adults with generalized anxiety disorder but only in the presence of negative emotional distractors [5].

Although the AX-CPT has been utilized widely with adults [13–16], relatively few studies have used this task with children [17–22]. The few available studies suggest that, beginning at age 6, children employ both proactive and reactive strategies [18, 19]. However, younger children engage proactive processes to a lesser extent and tend to rely more heavily on reactive control [18, 19] in part due to limited working memory capacity [17, 20, 22]. Critically, this research is limited in two key respects: (1) studies only examine children under the age of 11; (2) task complexity taxes children’s rule-learning abilities, often leading to task modifications across ages (See Supplement for information on common task modifications in children). These modifications complicate comparisons across studies and age groups. Thus, the development of proactive and reactive control strategy use remains largely unknown.

In children, anxiety emerges at ages that we know cognitive control is still developing [22–24]. At present, no studies test how childhood anxiety and strategy use (proactive vs. reactive) interact. However, White et al. (2017) demonstrated that high reactive control^1^ was associated with high fear in young children but that high reactive control was associated with *low* fear in older children. Other related findings show age to moderate the relation between childhood anxiety and error processing during a Flanker task [25, 26]: older children with anxiety exhibited increased neural response to errors; younger children exhibited a decreased response. Since these studies rely exclusively on tasks that measure reactive control, it remains unclear how anxiety in children relates to performance on tasks that provide contextual information to engage proactive control. Thus, there is need for work examining pediatric anxiety and proactive/reactive control over a large age-range.

The primary goal of the current study is to generate data comparing performance on the AX-CPT in youth with and without anxiety disorders. The secondary goal is to examine how age moderates anxiety-related performance on the AX-CPT in youth. To address these goals, the current study uses a child-friendly version of the AX-CPT (modeled after [11]) to evaluate associations between proactive and reactive cognitive control in a sample of children (*n* = 56), aged 8–18 with and without anxiety disorders. In line with prior studies examining wide-age ranges and responding during reactive control tasks [4, 25, 26], we hypothesized that the relation between anxiety and reactive strategy use will vary by age.

## Methods

### Participants

Sixty-nine children between the ages of 8 and 18 (M = 13.69 years, 18 male) participated in this study. This sample was identified via community outreach events and includes treatment-seeking youth with a primary diagnosis of an anxiety disorder (*n* = 42) and youth with no psychiatric diagnosis (*n* = 27; See Table 1 for sample demographics; See Supplemental Table S1 for details on the age distribution in both the anxious and healthy samples). Exclusionary criteria included: IQ < 70, history of significant mental illness (other than anxiety) or trauma, use of any psychoactive drugs within three months of participation. Of note, these exclusion criteria mirror those used in treatment studies of pediatric anxiety [27]. With this approach, findings generalize to similar samples of treatment-seeking anxiety disorder cases. Prior to participation, diagnostic status was confirmed by a doctoral- or master’s-level clinician (reliability k > 0.7) using the Schedule for Affective Disorders and Schizophrenia for School-Age Children-Present and Lifetime version (KSADS-PL; Kaufman et al. [28]). All diagnoses were then confirmed by a Board-Certified child and adolescent psychiatrist (DSP). Of note, 2 anxious individuals presented with ADHD (See supplement for analyses controlling for ADHD).

**Table 1:** Sample demographics (M(SD)) by anxiety diagnosis

Characteristic	ANX (n = 34)	HV (n = 27)	*t*	p value
Female(#)	24	19	0.0003^a^	0.99
Age (years)	13.52 (2.82)	13.92 (3.02)	− 0.53	0.596
IQ (WASI)	113.59 (10.83)	110.88 (14.06)	0.84	0.40
SCARED Total-child	28.69 (2.41)	10.69 (10.79)	5.35	< 0.001**
SCARED Total-parent	26.10 (9.84)	7.34 (8.98)	7.46	< 0.001**
SCARED Total-average	27.34 (9.73)	9.02 (8.38)	7.57	< 0.001**
PARS	14.68 (2.77)	1.95 (4.35)	13.4	< 0.001**

*ANX* anxious group, *HV* healthy volunteer group

^a^This value is a chi-squared statistic rather than *t* statistic

Parents identified their children as falling into the following racial groups: 65.6% White, 16.4% multiracial, 8.2% African American, 8.2% unknown, and 1.6% Native American or Alaskan Native. All study procedures were approved by the National Institute of Mental Health Institutional Review Board. Parents provided written informed consent and youth provided written assent prior to participation. Compensation for participating was $15.

### Data Loss

Of the 69 children that participated, 8 children’s data were excluded from the final sample because of technical error (*n* = 2), failure to finish the task (*n* = 5) or practice session (*n* = 1). Thus, 61 of 69 (88%) children provided complete AX-CPT data. Consistent with previous reports [22, 29] and to minimize the impact of preservative responding, we additionally excluded children with < 60% accuracy on BY trials (*n* = 5; 4/5 were under 14 years of age, 3/5 were anxious children) leaving 56 children with behavioral data that exceeded the accuracy requirement.

Additionally, 2 families elected to not complete any anxiety symptom questionnaires and 1 parent failed to fill out the Screen for Child Anxiety Related Emotional Disorders (SCARED). Thus, a total of 58 participants provided both parent- and self-report data. In the analyses reported below, when evaluating anxiety and age differences we include any participants who had data available.

### AX-CPT

The AX-CPT was administered on a Dell computer using E-prime 2.0 Professional and EyeLink 1000 Plus eye-tracking camera. Details about the eye-tracking administration and exploratory pupillometry analyses are presented in the Supplemental Materials.

During the AX-CPT, children were presented with a continuous stream of letters (presented one at a time) and asked to press a button for each letter presented (See Fig. 1). As has been reported previously in a similar age group (see [11]), to help the child track each pair, the first letter of the pair (the cue) was presented in light blue and the second letter in the pair (the probe) was presented in white. Children were asked to look for the letter pair A followed by X and were instructed to press 2 when they saw the “A” and 3 when they saw an “X” of the AX-pair. For all other letter pairs, the child was to press 2 for any non-A and 2 again for any non-X letter. These instructions ensured that AX trials (i.e., target trials) had a different response (i.e., 2 followed by 3) than all other trial types. Trials that comprised of non-A and non-X letters were categorized based on whether they had similarity to the target (i.e., AX pair). Following standards in the AXCPT literature, trials that involve a non-A cue were classified with a “B” and trials that involve a non-X probe were classified with a “Y.” Thus, a trial that involved an A cue but non-X probe were categorized as “AY” trials. Trials with a non-A cue but X-probe were categorized as “BX” trials. Lastly, trials that comprised of a non-A cue and non-X probe were deemed “BY” trials (See Fig. 1).

To begin, children received a practice phase during which participants received feedback on their performance on 2 AXCPT blocks (8 trials per block) to ensure they understood the target pair. After the practice blocks, no feedback was given as participants completed 3 additional blocks (50 trials per block) each with 70% AX trial types and 10% AY, 10% BX, 10% BY trial types. All letter pairs were presented in pseudorandom order. In total, participants completed 150 trials.

Stimulus presentation was consistent with previous childhood studies with the AX-CPT [11] with a few minor adjustments that facilitated the examination of pupil dilation following the presentation of both the Cue and Probe. Letter stimuli were presented in the center of the screen in Courier New, size 60 font. All stimuli were presented on a gray background. Each trial began with a centrally located fixation asterisk (the presentation was jittered between 200 and 500 ms) followed by the presentation of the cue stimulus (500 ms) with a 1000 ms response window (central fixation asterisk). Following the response window, there was a 3900 ms delay before the presentation of the probe (500 ms) and the following response window (1700 ms; See Fig. 1).

#### Expectations About Use of Proactive/Reactive Control by Trial Type

Distinguishing these trial types (AX, AY, BX, BY) allowed for the delineating of relative use of proactive and reactive control. In searching for this frequent stimulus pair (AX), distractor pairs provide information that allow the participant to proactively anticipate interference (e.g., a cue-probe pair: “B” followed by “X”). In this case, the B-cue indicates the absence of the “A” required for the AX target pair. Thus, an individual utilizing proactive control should respond relatively quickly and without error to BX trials because the B-cue provides the information needed to make a decision for how to respond to the probe. However, for this B-X pair, if the participant was relying on reactive control, they might see the X and need to think back to remember whether the cue was an A or non-A letter. In trying to quickly make this decision, participants may be more likely to mistakenly indicate that this is an AX-pair. On other trials, participants see pairs with similar cues but different probes (i.e., “A-Y” cue-probe pair), thereby requiring a different, immediate response to the probe. In the “A-Y” cue-probe pair, the probe (“Y”) conflicts with the expectation that A-cues are followed by X-probes. As such, an individual using proactive control will need to take longer in deciding their response to this unexpected probe in order to be accurate or may be more likely to mistakenly indicate that an AY-trial is actually the target trial type AX. On the flip side, individuals using primarily reactive control might do well on AY-trial types because they are attending more closely to the probe (“Y”) which is not an X and therefore doesn’t require thinking back to remember the cue. Still other trials, BY trial types, present no conflict as neither the A nor X is present. While these trials each provide unique information, the participant could respond to some trials reactively and others proactively. Thus, proactive/reactive strategy use is quantified by comparing performance across trial types.

#### Behavioral Data Pre-Processing

We computed accuracy for each trial type and overall. Accuracy was defined as a correct response to both the cue and probe. Split-half reliability estimates for accuracy were AX: *r* = 0.93, 95% CI [0.89 0.96], AY: *r* = 0.50, 95% CI [− 0.67 0.1], BX: *r* = 0.69, 95% CI [− 0.56 1], BY: *r* = 0.29, 95% CI [− 0.01 0.54]. We also extracted participant reaction times (RT) for all correct trials. Following conventions in the literature, we excluded all RTs that exceed 3 SDs from each individual’s overall mean RT (< 2% of trials). Average RTs for correct trials of each type were then transformed with respect to each subject’s grand mean RT over all correct trials. This approach has been utilized [18] to increase power and control for individual differences in overall processing speed. Split-half reliability estimates for reaction time were AX: *r* = 0.98, 95% CI [0.97 0.99], AY: *r* = 0.71, 95% CI [0.59 0.81], BX: *r* = 0.80, 95% CI [0.72 0.86], BY: *r* = 0.91, 95% CI [0.85 0.95]. See Supplemental Materials for average reaction times without correction for grand mean RT and for details on how reliability estimates were computed.

Using the accuracy data, we additionally computed d’prime and A-cue bias, two behavioral summary indices deriving from signal detection theory and commonly referenced in the AX-CPT literature [11, 12, 30, 31]. D’prime indexes sensitivity to cue information (i.e., how much the cue is used to inform future responses). D’prime is computed by subtracting incorrect responses on BX trials (false alarms) from correct responses on AX trials (hits). Higher d’prime scores indicate more planful strategy (consistent with proactive control) because it suggests that the participant is utilizing cue information to inform subsequent responses.

In contrast, the A-cue bias index provides an estimate of how likely a participant is to prepare a response based on the A cue (i.e., it measures a response bias for A). A-cue bias is computed identically to the c’ criterion measure of signal detection theory [32] and reflects criterion shift, given by the midpoint between the rate of AY false alarms and AX hits. Specifically, it is computed by summing hits on AX trials and false alarms on AY trials, then dividing by 2 (See [19]). Higher A-cue bias scores indicate more stringent criterion for responding that the probe was a target following an A-cue, consistent with greater proactive control. Lower A-cue bias scores indicate less stringent criterion for responding accurately to the probe in the presence of the A-cue, consistent with a less proactive pattern of responding.

### Symptom Measures

#### Screen for Child Anxiety Related Emotional Disorders (SCARED)

Anxiety symptoms were assessed using the Parent- and Child-Report versions of the Screen for Child Anxiety Related Emotional Disorders (SCARED [33]). The SCARED is comprised of 41 items presented on a 3-point Likert scale (0 = never/hardly ever true, 1 = sometimes/somewhat true, 2 = very/often true). Total scores were computed by summing across all items. Parent-Report scores ranged from 0 to 45 (M = 18.02, SD = 13.27) and Child-Report scores ranged from 0 to 55 (M = 21.063, SD = 15.53). Total score on the parent and child reports were significantly correlated (*r*(56) = 0.613; *p* < 0.0001). Age was not correlated with either parent or child report (*p* > 0.235). SCARED scores were summarized by taking an average of parent-report and child-report. See Supplemental Materials for results from parent- and child-report separately. Given the small sample size, the analyses below make use of continuous estimates of anxiety rather than diagnoses to increase power. See Supplemental Materials for exploratory analyses of group differences.

### Data Analytic Plan

#### Accuracy and RT

Given the wide age range, we first tested whether age exhibited expected correlations with accuracy and RT. Next, we evaluate effects of age (measured continuously), anxiety symptoms (measured via SCARED-average parent and child total score) and their interaction on accuracy and standardized reaction time (RT) by trial type (AX, AY, BX, BY). To do so, we ran repeated measures ANOVAs with age and anxiety symptoms as continuous between-subjects factors and trial type (AX, AY, BX, BY) as the repeated measures factor.

#### Behavioral Summary Measures (d’prime and A-cue bias)

To evaluate the relation between age, anxiety and individual differences in the extent to which participants used the cue to inform their subsequent responses, we conducted an univariate ANOVA with age and anxiety as continuous between-subjects factors and d’prime as our dependent variable. Additionally, to test whether the results of our d’prime model could be explained by a response bias to the A cue, we conducted a separate univariate ANOVA to evaluate the effects of age and anxiety on A-cue bias. See Supplemental Table S2 for correlations among key variables in these models.

## Results

### Accuracy and RT

**Fig. 2:**
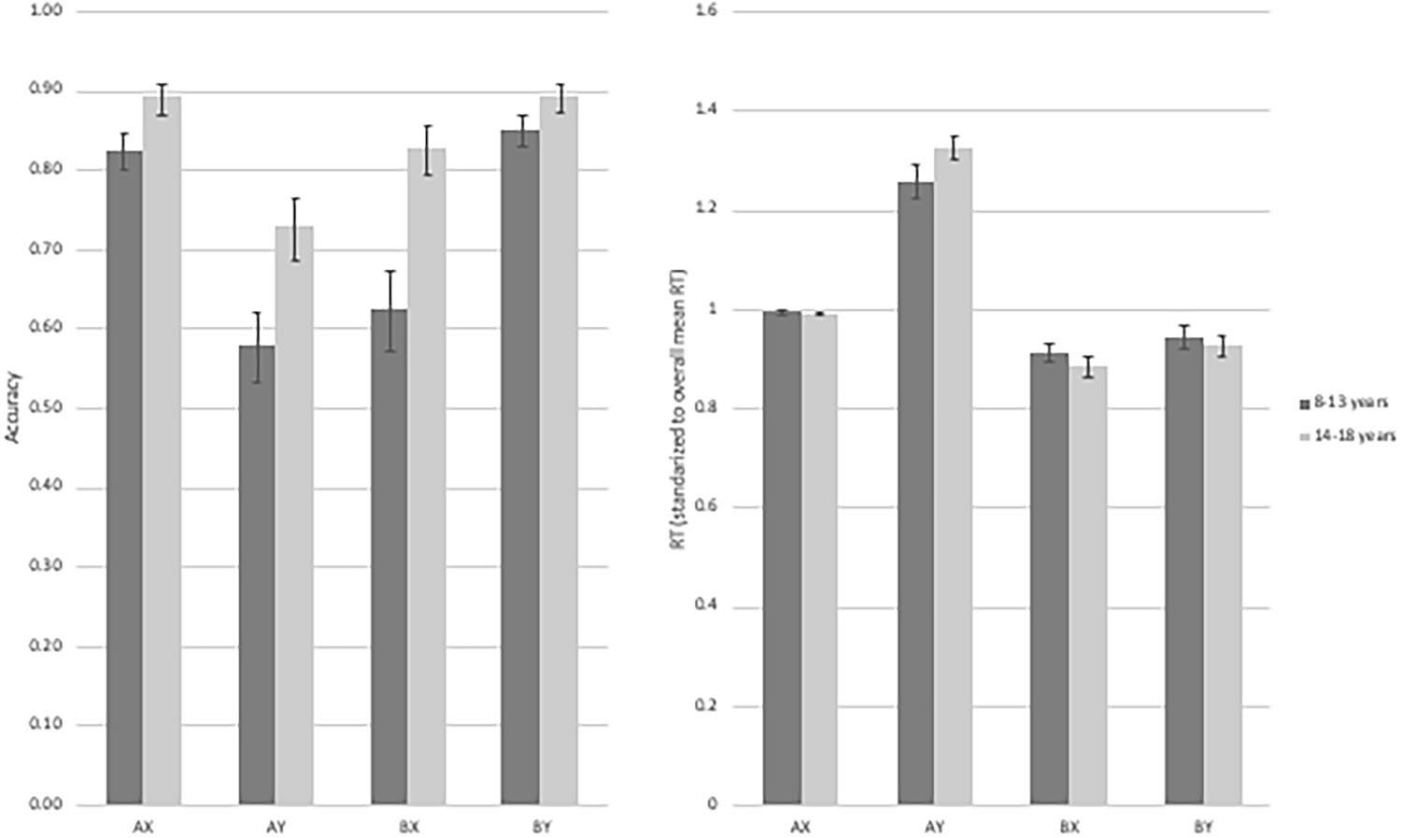
Illustrates accuracy (left) and reaction time (RT; right) as a function of age (*N* = 56). Age is divided via median split (Median = age 13) for illustrative purposes only. All analyses were conducted using age as a continuous variable

As expected, for overall task performance, older children exhibited greater accuracy (*r*(54) = 0.398, *p* < 0.002) and were faster at responding (*r*(54) =  − 0.416 *p* < 0.001) than younger children. Next, we evaluated the effect of age and anxiety on accuracy across specific trial types (AX, AY, BX, BY; See Fig. 2). Mauchly’s test indicated that the sphericity assumption was violated for both the accuracy (*X*(5) = 64.350, *p* < 0.001) and reaction time (*X*(5) = 20.788, *p* < 0.001) models. Thus, degrees of freedom were corrected Greenhouse–Geisser estimates (ℇ_accuracy_ = 0.633; ℇ_RT_ = 0.811). Results for accuracy revealed a significant age-by-anxiety interaction (*F*(1,50) = 5.564, *p* < 0.022, ηp^2^ = 0.100), indicating that among those with high anxiety, older children were significantly more accurate overall than younger children (association between age and overall accuracy: *r*(31) = 0.448, *p* < 0.011). No age differences in accuracy were found among children low on anxiety. Also, older, high anxious and low anxious children did not differ from each other in accuracy. Results further indicated a significant trial type-by-anxiety interaction (F(1.898, 94.894) = 3.282, *p* < 0.044, ηp^2^ = 0.062). Follow-up analyses indicated that high anxious individuals showed the lowest accuracy on BX trials. Results for RT revealed neither main effects nor interaction effects (*p*s > 0.211).

### Behavioral Summary Measures (d’prime & A-Cue Bias)

**Fig. 3:**
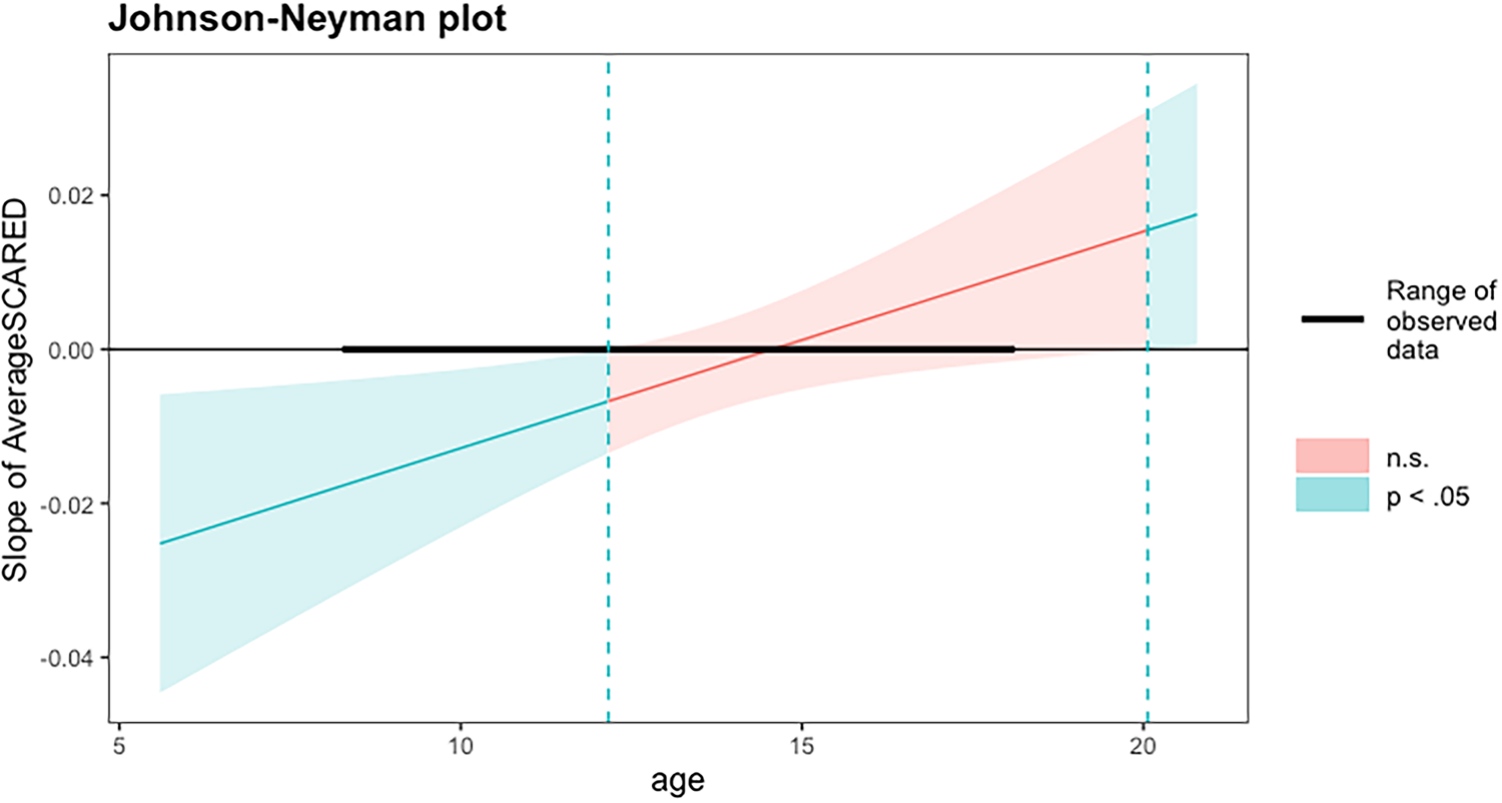
Illustrates the conditional slope of average SCARED. Blue highlighted line indicates the ages at which the relation between anxiety and d’prime is significant, red highlighted line indicates ages where this association is not significant. Black bar indicates range of observed ages (i.e., age 8–18). See Supplement for figure illustrating the raw data and linear fit when age bins are created using mean ± 1 standard deviation

The next analyses assessed individual differences in the tendency to use cue information to inform subsequent responses (d’prime) and in levels of response bias for probe targets following the A cue (A-cue bias). To do so, we analyzed our behavioral summary measure data. Results indicated that there was an age-by-anxiety interaction on d’prime (*F*(1,50) = 6.175, *p* < 0.016, ηp^2^ = 0.110). To probe the interaction, a simple slopes analysis (using Johnson-Neyman intervals) was conducted to identify the conditional slope of the average SCARED at different ages. This analysis approach allows us to identify the ages at which the association between d’prime and average SCARED is significant without making arbitrary age cut-offs (although see supplement for report of the data binarized using mean ± 1 standard deviation). Results indicated that there was a significant negative association between anxiety and d’prime in younger children (i.e., those between ages age 8 and 12) but no significant association between anxiety and d’prime in older children (those children between age 12 and 18; See Fig. 3). Thus, among younger children, those with greater anxiety exhibit less planful behavior, consistent with less proactive strategy use. Critically, results further indicated that there were no significant age, anxiety or interaction effects of A-cue bias (*p*s > 0.502)—suggesting that these effects are not explained by a response bias to the A cue. Although, given the sample size, these results should be interpreted as preliminary and require further replication.

## Discussion

The current study reports the first data on AX-CPT performance in anxious and non-anxious children. Findings provide novel insights on age-related variation in the association between cognitive control and anxiety. Young children with high anxiety exhibit less planful behavior, consistent with less proactive strategy use, than age-matched youth with low anxiety. Critically, this association cannot be explained by a heightened response bias to the cue. These differences in cognitive control strategy use are paralleled by age-by-anxiety interactions in overall accuracy. Among highly anxious children, older children responded more accurately than younger children; this age-related pattern was not found in low anxious children. Together these findings suggest that younger children with anxiety exhibit less planful behavior than age-matched children with low anxiety. Overall, of note, these results should be considered preliminary, given small sample sizes. However, the work does extend past research on pediatric anxiety.

Rumination and worry in anxious individuals is thought to degrade the ability to maintain goals (a process that is required for executing proactive control), thereby creating a need for deployment of compensatory reactive control [1, 2]. Various studies have examined how individuals with anxiety disorders perform on reactive-control tasks [3, 4, 9, 10, 26, 34–41]. However, only one study has tested clinically anxious individuals using a modified version of the AX-CPT which included embedded emotional stimuli [5].^2^ Of note, many prior studies utilize other cognitive tasks with such embedded emotional stimuli. While such tasks directly examine relations among anxiety, emotional content, and cognitive processes, these tasks may fail to accurately assess cognitive performance in the absence of emotional stimuli. Moreover, questions remain on the generalizability of findings from studies using emotional stimuli to more frequently-used versions of the AX-CPT, free of emotional content [5]. The current study utilized the more frequently-used, non-emotional version of the AX-CPT task, so that our results could be compared with this broader literature. Nevertheless, findings from the one study using an emotional version of the AX-CPT [5] are relevant to our findings, as this prior study suggests that anxious individuals may exhibit improved cognitive control under at least some circumstances. In line with this conclusion, our sensitivity analyses (See Supplement) demonstrate that older children who use the cue to guide their subsequent behavior (a strategy consistent with proactive control) have higher parent-reported anxiety. This pattern was not found with child-report or younger children. Future studies with large sample sizes are needed to assess the reliability of these effects.

Proactive and reactive processes are recruited on different time scales—proactive processes are recruited when anticipating future events; reactive processes are recruited when the events occur. The current findings suggest that younger relative to older children with high levels of anxiety may be less adept at employing a proactive anticipatory strategy. This is broadly consistent with longitudinal studies among individuals at temperamental risk for anxiety, where greater reactive strategy use relates to risk for anxiety [44, 45]. Similarly, other data suggest that increases in inhibitory control from age 5–10 years predicts greater anxiety [46]. Speculatively, since cognitive control resources are capacity-limited, heightened error monitoring and inhibitory control could deplete these resources. This in turn could impair children’s ability to recognize the relevance of preparatory cue-based contextual information pertinent to upcoming probes. Our findings suggest that these effects could be particularly marked for young children with high anxiety. Nevertheless, other factors to be targeted in subsequent research could account for our age-related findings. These might include specific maturational processes or the age of anxiety onset. Identifying these factors may inform individualized treatment strategies.

Cognitive control plays a complex role in anxiety—particularly in pediatric anxiety. Childhood involves maturational changes in the prefrontal cortex which supports cognitive control [23, 47] and relate to changes in response inhibition, one component of cognitive control [48, 49]. However, to date, little work has examined cognitive control processes in anxious youth. Research that has directly tested age-by-anxiety interactions found age differences in the relation between childhood anxiety and error monitoring [25, 26]. The findings reported here further suggest the importance of considering age moderation in the cognitive control-by-anxiety relation. Additional research on the role of prefrontal cortex function in such moderation could provide particularly helpful insights. Thus, research on the pathophysiology of pediatric anxiety may benefit from further research on age-related differences.

### Limitations

While this study has several strengths including its focus on dissociating proactive and reactive control in children, it also has several notable limitations. First, the sample size of the study was small (*n* = 56 both the anxious and non-anxious groups combined). At this sample size, we were powered at 0.80 to detect medium to large size effects (See Supplement). As a result, the current findings provide only one initial step in research on age moderation of the anxiety-cognitive control relation. Second, this study was cross-sectional rather than longitudinal. As such, we cannot know if the age-effects hold within individuals across time. Due to the small sample size and cross-sectional nature of these data, our age-effects should be viewed as preliminary in nature. Additional longitudinal studies using the AX-CPT are needed to determine whether the age of anxiety onset influences the development of control strategy use or whether there are changes within anxious individuals over time. Finally, the analyses reported here average parent and child report of anxiety symptoms. Averaging anxiety symptoms takes into account both reporters and may be more reliable over time. However, research has shown that parent report on the SCARED is a better predictor of children’s anxiety during structured tasks; whereas, child report on the SCARED is a better predictor of anxiety during naturalistic situations [50]. As such, averaging parent and child report may have limitations. Thus, we provide analyses examining parent- and child-report separately in the supplement. Additional studies are also needed to evaluate how strategy use is impacted by threat. Several studies demonstrate that anxious children show an attention bias to threat, that this bias is linked to symptom severity [51, 52], and that attention bias modification treatments can decrease anxiety symptoms [53, 54]. However, it remains unclear how attention bias to threat and cognitive control interact in anxious individuals (See [55, 56] for discussion of how these mechanisms may interact). Some anxious individuals only show impaired cognitive control in threatening contexts [57]. At present, it remains unclear how the current findings might generalize to such contexts, where the balance between reactive and proactive strategies may change [1, 56]. Future work should consider this possibility.

## Conclusion

In conclusion, these results provide novel evidence that the association between planful behavior and anxiety varies as a function of age. Young children with high anxiety exhibit less planful behavior, consistent with less use of proactive control. Critically, this is the first study to report the use of the AX-CPT in children with anxiety—thereby providing the first evidence of cognitive control *strategy use* in a pediatric anxious sample. These findings suggest that maturational changes shape strategy use in children with anxiety.

## Summary

Behavioral control can be instantiated either immediately (i.e., when a stimulus indicates that you need to respond) or in anticipation of an *upcoming* event/stimulus that will require control. This difference in the timing of control processes is referred to as the reactive (i.e., stimulus-drive) vs. proactive (i.e., anticipatory/preparatory) control. Anxiety is thought to be associated with a reliance on reactive control. However, at present, few studies utilize tasks that dissociate the use of reactive vs. proactive cognitive control strategies in response to conflict, and none examine children diagnosed with anxiety. The current study utilizes the AX-CPT, a cognitive control task which dissociates reactive and proactive control, to examine cognitive control in youth (age 8–18) with and without an anxiety diagnosis (*n* = 56). While treatment-seeking anxious and healthy children were recruited, anxiety was operationalized dimensionally using the Screen for Child Anxiety Related Emotional Disorders (SCARED). Analyses examined the effects of age and anxiety on cognitive control strategy use. Results showed a significant interaction between age and anxiety on strategy use. Specifically, we found that young children with high anxiety exhibit significantly less planful behavior, consistent with less proactive strategy use, compared to similarly-aged children with low anxiety. This pattern was not found in older children. These findings highlight the importance of considering how maturation influences the impact of anxiety on behavioral performance.

## Supplementary Information

Below is the link to the electronic supplementary material.

Supplementary file1 (DOCX 324 KB)

## Data Availability

The data that support the findings of this study are available from the corresponding author (Courtney Filippi, PhD) upon reasonable request.

## Footnotes

1 In this study, White et al. (2017) made use of bifactor modeling of cognitive control. The results summarized here come from her response inhibition factor which was comprised of Go/NoGo behavioral performance.

2 Although see Yang et al. [43] for study on how state anxiety influences performance on the AX-CPT and see Krug and Carter [42] for relations to trait anxiety.
