# Supplementary material for: Developmental Changes in the Association Between Cognitive Control and Anxiety
Source: Child Psychiatry Hum Dev. 2021 Mar 18;53(3):599–609. doi: 10.1007/s10578-021-01150-5 (PMC9107422; doi:10.1007/s10578-021-01150-5)
Supplement: Supplementary file 1 — Supplementary file1 (DOCX 324 KB) [file 10578_2021_1150_MOESM1_ESM.docx]

**Supplemental Materials**

**Common task modifications for Children.** As indicated in the main text, several studies in the literature modify the AX-CPT for children. This makes comparison across studies difficult. Common modifications for children (used in prior studies but not the current study) include: eliminating responding to the cue thus necessitating only a single response to the probe, narrowing the delay period between the cue and probe, hanging stimuli from letters to child-friendly images, changing task instructions from pairings to preferences (e.g., Nemo likes blackberries), elongating the response window, and making the response window adaptive.

**Age of Sample Binned by year.** In Table S1, we provide additional information about the age distribution of both the anxious and healthy samples. As you can see from Table S1, the age distribution is comparable for both anxious and healthy participants.

Table S1. Age distribution in the anxious and healthy sample (ages in years).

| age  (in years) | Anxious | Healthy |
| --- | --- | --- |
| 8 | 0 | 1 |
| 9 | 3 | 3 |
| 10 | 2 | 2 |
| 11 | 3 | 1 |
| 12 | 2 | 1 |
| 13 | 5 | 3 |
| 14 | 5 | 3 |
| 15 | 3 | 4 |
| 16 | 3 | 3 |
| 17 | 4 | 4 |
| 18 | 1 | 0 |

**Correlations Among Key Variables.**

Table S2. Correlations between sample characteristics and summary measures of reactive/proactive control.

| Measure | (1) | (2) | (4) | (5) | (6) |
| --- | --- | --- | --- | --- | --- |
| 1. Age | -- | -- | -- | -- | -- |
| 2. SCARED Average | -.023 | -- | -- | -- | -- |
| 3. IQ | -.142 | -.012 | -- | -- | -- |
| 4. d-prime | .394** | -.109 | -.016 | -- | -- |
| 5. A-Cue Bias | -.185 | -.005 | .006 | -.028 | -- |

**Reliability of reaction time measures.** Permutation-based split-half reliability estimates were obtained for each trial type using the splithalf package in R (v.7.1, Parsons, 2020). Results are averaged over 5000 random splits. Estimates of reaction time were computed for accurate trials only. For estimates of reaction time reliability, subjects needed to contribute at least 4 accurate trials to be included in the analysis. This resulted in the exclusion of *n*=2 for AY estimates, and *n*=3 for BX estimates. Reliability estimates reported in the main text are Spearman-Brown corrected and include 95% confidence intervals.

**Power of D’prime Results.** With a sample size of n=56, this study was equipped to detect medium to large effect sizes (f^2^>.22) with power of over .80 at an alpha level .05. Results in the main text demonstrated that there was a significant age-by-anxiety interaction on d’prime. With a sample size of 56 and effect size of ηp^2^=.11 (for the interaction), the observed power is .68.

**Interaction plotted at mean age +/- 1 standard deviation.** In the main text, we make use of simple-slopes analysis using Johnson-Neyman intervals to determine the precise age at which the association between anxiety and d’prime is significant. Results of this analysis indicated that the association between anxiety and d’prime was significant from 8 -12 years of age but not in children over the age of 12. However, another common method of visualizing interactions is to plot the raw data at set age groupings. In the figures below we present the raw data and linear fit at the mean age (13.88 years) and +/- one standard deviation (16.61 years and 11.15 years, respectively). Results indicated that the only significant slope was that of the -1 standard deviation group (i.e., the younger children, β=-.01, *p*<.02). See Figure S1 below.


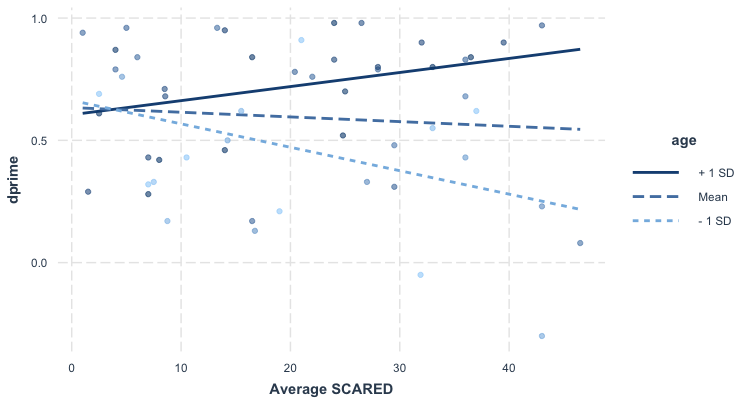


Figure S1. Illustrates the association between average SCARED and d’prime at the mean age (13.88 years) and at +/- 1 standard deviation (ages 16.61 years and 11.15 years, respectively). Dots depict raw data. Lines depict linear fit.

**Controlling for ADHD.** Very few anxious children in the sample met criteria for ADHD, since the presence of ADHD requiring immediate treatment was an exclusion criterion. Nevertheless, it is important to consider the potential influence that ADHD symptoms could have on the results. In addition to assessing ADHD via clinical interview, the Conners was administered to estimate ADHD symptoms continuously (*n*=52; *M*=7.19, *SD*=7.07). When comparing the anxious and healthy samples, there was a significant difference in Conners scores—with anxious children (*M*=9.76) scoring significantly higher (*t*(50)=3.196, *p*<.002) than healthy control children (*M*=3.96).

In this supplemental analysis, we examine whether accuracy and RT results are maintained when covarying for ADHD symptoms as measured via the Conners. Results of the accuracy model revealed that the age-by-anxiety interaction remained significant (*p*<.047) after controlling for ADHD symptoms. However, the significant trial type-by-anxiety interaction was no longer significant (*p*>.104). As in the main text, the RT model showed no main effects nor any interaction effects after controlling for ADHD symptoms.

In this supplemental analysis, we examine whether the d’prime and A-cue bias results are maintained when we covary for ADHD symptoms (measured via the Conners). Results demonstrated that the age-by-anxiety interaction on d’prime remained marginally significant (*p*<.053) when controlling for ADHD symptoms. Additionally, there was a significant main effect of anxiety (*p*<.033) but no main effect of age. Further, as in the main text, the A-cue bias model showed no significant main effects or interactions when controlling for ADHD symptoms.

**Sensitivity Analyses: SCARED-parent and SCARED-child separately.** In the main text the focal analyses examine whether the relation between anxiety and proactive/reactive strategy varies by age. However, notably anxiety was operationalized as the average of parent report and child-report on the SCARED. Some studies suggest that averaging parent and child report can be problematic. As such, in this section we re-run our focal analyses for child-report and parent-report separately.

Results indicated that the association between child-reported anxiety and d’prime differed as a function of age (*F*(1,50)=5.244, *p*<.026). Specifically, there was a significant negative association between anxiety and d’prime in younger children (*r*(23)=-.448, *p*<.025) but no significant association between anxiety and d’prime in older children (*r*(27)=.224, *p*<.242). These results converge with the main text.

Results indicated that the association between parent-reported anxiety and d’prime differed as a function of age (*F*(1,49)=5.385, *p*<.025). Specifically, there was no significant association between anxiety and d’prime in younger children (*r*(22)=-.377, *p*<.070) but there was a significant positive association between d’prime and parent-reported anxiety for older children (*r*(27)=.420, *p*<.023). These results suggest differ from the main text and suggest that older children who exhibit more planful behavior (a pattern consistent with greater proactive control) exhibit greater parent-report of children’s anxiety. These results hint at an age-related difference in the association between parent-reported anxiety and planful behavior.

**Modeling by diagnostic group.** In the main text, to increase power, we decided to examine effects of anxiety using continuous scores on the SCARED. However, given that the sample was recruited based on diagnoses, here we reproduce the models in the main text using diagnostic group (anxious vs. healthy controls) rather than SCARED scores in the model.

**Accuracy and RT.** Results of the accuracy model demonstrated that accuracy differed as a function of trial type (*F*(1.91,99.33)=5.525, *p*<.006) and age (*F*(1,52)=12.365, *p*<.001). No other main effects or interactions were found. Results of the reaction time model demonstrated that reaction time differed as a function of age (*F*(1,50)=8.29, *p*<.006). No other main effects or interactions were found.

**D’prime and A-cue Bias.** Results of the d’prime model demonstrated a main effect of age (*F*(1,52)=10.23, *p*<.002) but no other main effects or interactions. Results of the A-cue bias model demonstrated no significant main effects nor interactions.

**Pupillometry.**

Given our limited understanding of performance on the AX-CPT across this wide age range, in addition to behaviorally assessing performance, we concurrently assessed pupillometry. Pupillometry is a method that measures the size of the pupil over time and provides an implicit metric of arousal/effort and attention [1,2]. Increased pupil size during the delay between the cue and probe (particularly to nontarget cues) and following the probe (on trials where the prepotent response had to be overcome) has been utilized as an index of proactive/reactive control in children [3] and adults [4,5]

**Data acquisition.** Participants underwent a standard eye-tracking calibration procedure during which they sat in a fixed chair within 520 mm from the desktop mounted eye tracking camera. Eye level was consistent across all participants and real-time binocular eye movement data was captured continuously throughout the task at 1000 Hz per second.

**Data Loss.** An additional four participants were excluded from pupillometry analyses because of technical failures, leaving 52 children who had both usable eye-tracking and behavioral data. In the analyses reported below, when evaluating group and age differences we include any participants who had useable data available.

**Analytic Methods.** All eye-tracking data was extracted and loaded into TimeStudio v.3.18 [6], a Matlab plugin. Data were collected at 1000 Hz, indicating that there were 1000 samples collected per second. Trials were excluded if 75% or more of the samples collected in a given trial were missing. To reduce noise and smooth the signal, a sliding-window average (5 samples wide) was applied at each time point. Data were then detrended and z-scored. The baseline was selected as -200 ms to 0 relative to the start of either the cue or probe. This corresponds to the minimum amount of jitter between trials. In line with prior work, we next visually inspected the average time series from -1000 ms prior to the presentation of the stimulus (Cue or Probe) to 3500 ms post-Cue and 2200 ms post-Probe presentation^[[1]](#footnote-1)^. We then extracted the average (baseline corrected) pupil response from the time period immediately following the light reflex through to the end of the trial (1500-3500 ms following the presentation of the cue and 1500-2200 ms following the presentation of probe) for all trials of each trial type. The full time series for all trial types are plotted below. Data were time-locked to both the cue (A or B) and the probe (AX, AY, BX, BY).

**Data Analysis Approach**. Finally, we assess whether pupil dilation to the cue or probe differed as a function of age and/or anxiety. To do so, we ran two separate repeated measures ANOVAs (model 1: time-locked to the cue; model 2: time-locked to the probe) with age and anxiety as between-subjects factors and trial type (AX, AY, BX, BY) as a repeated measures factor.

**Results.** Results indicated that there were no significant main effects or interactions to the probe-locked pupil response (*p*s>.169) or cue-locked pupil response (*p*s> .259; See Figures S2 and S3 for time courses). These results demonstrate a failure to replicate prior studies, albeit using a more simple version of the task, administered to younger children [3]. These time courses also suggest potential global age-related differences in pupillometry.


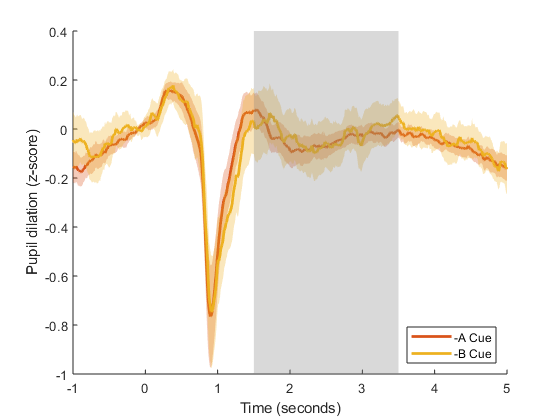


Figure S2. Pupillometry time course locked to the cue.


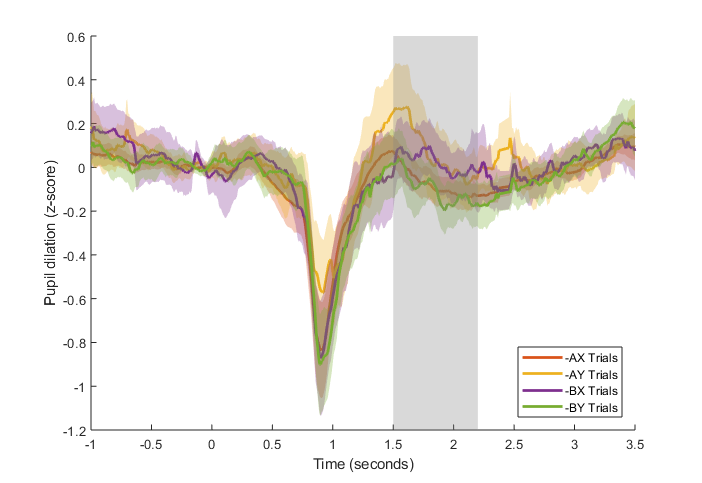


Figure S3. Pupillometry time course locked to the probe.

References

[1] Laeng B, Sirois S, Gredebäck G. Pupillometry: A Window to the Preconscious? Perspect Psychol Sci 2012;7:18–27. https://doi.org/10.1177/1745691611427305.

[2] Piquado T, Isaacowitz D, Wingfield A. Pupillometry as a measure of cognitive effort in younger and older adults. Psychophysiology 2010;47:560–9. https://doi.org/10.1111/j.1469-8986.2009.00947.x.

[3] Chatham CH, Frank MJ, Munakata Y. Pupillometric and behavioral markers of a developmental shift in the temporal dynamics of cognitive control. Proc Natl Acad Sci 2009;106:5529–33. https://doi.org/10.1073/pnas.0810002106.

[4] Chiew KS, Braver TS. Dissociable influences of reward motivation and positive emotion on cognitive control. Cogn Affect Behav Neurosci 2014;14:509–29. https://doi.org/10.3758/s13415-014-0280-0.

[5] Mäki-Marttunen V, Hagen T, Aminihajibashi S, Foldal M, Stavrinou M, Halvorsen JH, et al. Ocular signatures of proactive versus reactive cognitive control in young adults. Cogn Affect Behav Neurosci 2018;18:1049–63. https://doi.org/10.3758/s13415-018-0621-5.

[6] Nyström P, Falck-Ytter, T, Gredebäck G. The TimeStudio Project: An open source scientific workflow system for the behavioral and brain sciences. Behav Res Methods 2016;48:542–52.

1. The differences in the Cue and Probe end-time was due to differences in the timing of the trial—there was a 3900 ms delay between Cue and Probe and only a 2400-2700 ms delay between the Probe presentation and the start of the next Cue. [↑](#footnote-ref-1)
